# (2*S*,5*S*,6*R*)-5-(4-Methyl­phen­yl)-3-phenyl-4,8-dioxa-3-aza­tricyclo­[7.4.0.0^2,6^]trideca-1(13),9,11-triene-6-carbonitrile

**DOI:** 10.1107/S1600536811055413

**Published:** 2012-01-07

**Authors:** K. Swaminathan, K. Sethusankar, J. Srinivasan, M. Bakthadoss

**Affiliations:** a Department of Physics, RKM Vivekananda College (Autonomous), Chennai 600 004, India; b Department of Organic Chemistry, University of Madras, Maraimalai Campus, Chennai 600 025, India

## Abstract

In the title compound, C_24_H_20_N_2_O_2_, the six-membered pyran ring adopts a half-chair conformation with one C atom deviating from the mean plane of the remaining ring atoms by 0.654 (6) Å. The five-membered isoxazole ring adopts an N-envelope conformation with the N atom displaced by 0.742 (5) Å from the mean plane formed by the remaining ring atoms. The carbonitrile side chain is almost linear, with a C—C—N angle of 178.6 (5)°. The crystal packing is stabilized by inter­molecular C—H⋯N inter­actions, through bifurcated acceptor hydrogen bonds formed between the carbonitrile N atom and two alternate C atoms in the unsubstituted benzene ring. The mol­ecular structure and crystal packing are further stabilized by intra­molecular and inter­molecular C—H⋯π inter­actions.

## Related literature

For uses of benzopyran and isoxazolidine derivatives, see: Green *et al.* (1982 ▶); Kashiwada *et al.* (2001 ▶); Mullen *et al.* (1988 ▶). For a related structure, see: Swaminathan *et al.* (2011 ▶). For puckering parameters, see: Cremer & Pople (1975 ▶). For synthetic details, see: Bakthadoss & Murugan (2010 ▶).
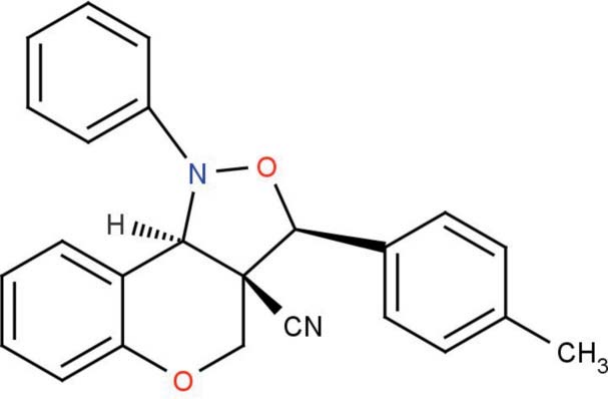


## Experimental

### 

#### Crystal data

C_24_H_20_N_2_O_2_
*M* *_r_* = 368.42
Orthorhombic, 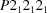

*a* = 8.9890 (16) Å
*b* = 9.8432 (19) Å
*c* = 22.084 (4) Å
*V* = 1954.0 (6) Å^3^
*Z* = 4
Mo *K*α radiation
μ = 0.08 mm^−1^
*T* = 293 K
0.30 × 0.25 × 0.25 mm

#### Data collection

Bruker Kappa APEXII CCD diffractometer
Absorption correction: multi-scan (*SADABS*; Bruker, 2008 ▶) *T*_min_ = 0.976, *T*_max_ = 0.980
10510 measured reflections
4476 independent reflections
2396 reflections with *I* > 2σ(*I*)
*R*_int_ = 0.065

#### Refinement

*R*[*F* ^2^ > 2σ(*F* ^2^)] = 0.081
*wR*(*F* ^2^) = 0.252
*S* = 1.00
4476 reflections
254 parameters
H-atom parameters constrained
Δρ_max_ = 0.29 e Å^−3^
Δρ_min_ = −0.33 e Å^−3^

### 

Data collection: *APEX2* (Bruker, 2008 ▶); cell refinement: *SAINT* (Bruker, 2008 ▶); data reduction: *SAINT*; program(s) used to solve structure: *SHELXS97* (Sheldrick, 2008 ▶); program(s) used to refine structure: *SHELXL97* (Sheldrick, 2008 ▶); molecular graphics: *ORTEP-3* (Farrugia, 1997 ▶); software used to prepare material for publication: *SHELXL97* and *PLATON* (Spek, 2009 ▶).

## Supplementary Material

Crystal structure: contains datablock(s) global, I. DOI: 10.1107/S1600536811055413/pv2496sup1.cif

Structure factors: contains datablock(s) I. DOI: 10.1107/S1600536811055413/pv2496Isup2.hkl

Supplementary material file. DOI: 10.1107/S1600536811055413/pv2496Isup3.cml

Additional supplementary materials:  crystallographic information; 3D view; checkCIF report

## Figures and Tables

**Table 1:** Hydrogen-bond geometry (Å, °) *Cg*1 and *Cg*2 are the centroids of the C19–C24 and C12–C17 rings, respectively.

*D*—H⋯*A*	*D*—H	H⋯*A*	*D*⋯*A*	*D*—H⋯*A*
C5—H5⋯*Cg*1	0.93	2.99	3.701 (5)	134
C20—H20⋯*Cg*2^i^	0.93	2.95	3.803 (6)	153
C21—H21⋯N2^ii^	0.93	2.56	3.385 (6)	148
C23—H23⋯N2^iii^	0.93	2.62	3.466 (7)	151

Symmetry codes: (i) 
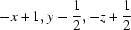
; (ii) 
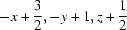
; (iii) 
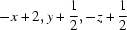
.

**Fig. 1.**
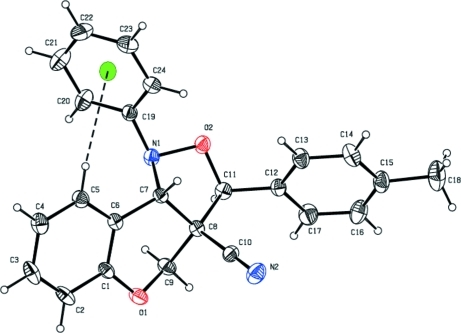
The molecular structure of the title compound with the atom numbering scheme, depicting the C—H···Cg intramolecular interaction as dashed line. Displacement ellipsoids are drawn at 30% probability level. H atoms are present as small spheres of arbitary radius.

**Fig. 2.**
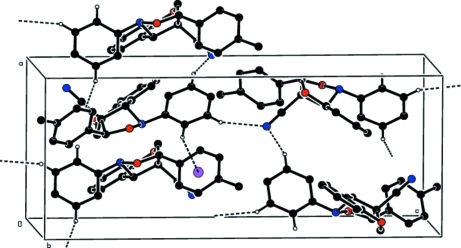
Part of the crystal structure of the title compound viewed down the a axis; C—H···N and C—H···Cg intermolecular interactions are indicated by dashed lines.

## supplementary crystallographic information

### Comment

There has been a flurry of activity on the synthesis of benzopyran and isoxazolidine derivatives owing to their well established biological and pharmacological activities. For example, some benzopyran derivatives exhibit anti-HIV activities (Kashiwada *et al.*, 2001). Similarly, isoxazolidine derivatives exhibit anti-fungal (Mullen *et al.*, 1988), anti-inflammatory (Green *et al.*, 1982) and other such biological activities. In this paper we report the synthesis and crystal structure of the title compound which comprises an isoxazole ring, *trans*-fused with the pyran ring of the chromene moiety, a methyl benzene ring and a carbonitrile group *cis*-attached to the adjacent carbon atoms and a benzene ring attached to the nitrogen atom of the isoxazole ring.

In the title molecule (Fig. 1), the six membered pyran ring adopts a *half-chair* conformation with puckering parameters (Cremer & Pople, 1975): *Q* = 0.487 (4) Å, θ = 54.1 (5)° and φ = 288.0 (5)°; the carbon atom C9 deviates from the mean plane by 0.654 (6) Å. The five membered isoxazole ring adopts an N-*envelope* conformation with N1 displaced by 0.742 (5) Å from the mean plane formed by the rest of the ring atoms. The carbonitrile side-chain is almost linear, with C–C–N angle of 178.6 (5)°, which is in agreement with the observations made in another similar reported structure (Swaminathan *et al.*, 2011).

The molecular structure is stabilized by a C—H···*Cg*1 intramolecular interaction (Fig 1); *Cg*1 is the center of gravity of the phenyl ring (C19–C24). The crystal packing is stabilized by two C–H···N intermolecular interactions, through bifurcated hydrogen bonds formed between the carbonitrile N atom and two alternate C atoms in the unsubstituted benzene ring and a C—H···*Cg*2 intermolecular interaction (Table 1); *Cg*2 is the center of gravity of the phenyl ring (C12–C17). The packing arrangement of the title compound is shown in Fig. 2.

### Experimental

The title compound was synthesized using Baylis–Hillman derivatives through *in situ* formation of nitrones followed by an intramolecular [3 + 2] dipolar cycloaddition reaction sequence (Bakthadoss & Murugan, 2010). A mixture of (*E*)-2-((2-Formylphenoxy)methyl)-3-*o*-tolylacrylonitrile (2 mmol, 0.75 g) and *N*-phenylhydroxylamine (3 mmol, 0.33 g) in ethanol (10 ml) was refluxed for 6 h. After the completion of the reaction, as indicated by TLC, the reaction mixture was concentrated and the resulting crude mass was diluted with water (15 ml) and extracted with ethyl acetate (3 *x* 15 ml). The combined organic layer was washed with brine (3 *x* 15 ml) and dried over anhydrous Na_2_SO_4_. The solvent was removed under reduced pressure. The crude mass was purified by column chromotography on silica gel (Acme 100–200 mesh), using ethyl acetate-hexane (1:9) to provide the pure title compound as a colourless solid in 80% yield. Block-shaped single crystals of the title compound suitable for X-ray diffraction analysis were obtained from a solution of ethyl acetate by slow evaporation at room temperature.

### Refinement

The hydrogen atoms were placed in calculated positions with C–H = 0.93, 0.96, 0.97 and 0.98 Å for aryl, methyl, methylene and methyne type H-atoms, respectively, and refined in the riding mode with fixed isotropic displacement parameters: *U*_iso_(H) = 1.5 *U*_eq_(C) for methyl and *U*_iso_(H) = 1.2 *U*_eq_(C) for other H-atoms. In the absence of significant anomalous dispersion effects, an absolute structure was not determined, and 1859 Friedel pairs were merged.

### Crystal data

**Table:**

C_24_H_20_N_2_O_2_	*F*(000) = 776
*M**_r_* = 368.42	*D*_x_ = 1.252 Mg m^−^^3^
Orthorhombic, *P*2_1_2_1_2_1_	Mo *K*α radiation, λ = 0.71073 Å
Hall symbol: P 2ac 2ab	Cell parameters from 4476 reflections
*a* = 8.9890 (16) Å	θ = 1.8–27.7°
*b* = 9.8432 (19) Å	µ = 0.08 mm^−^^1^
*c* = 22.084 (4) Å	*T* = 293 K
*V* = 1954.0 (6) Å^3^	Block, colourless
*Z* = 4	0.30 × 0.25 × 0.25 mm

### Data collection

**Table:**

Bruker Kappa APEXII CCD diffractometer	4476 independent reflections
Radiation source: fine-focus sealed tube	2396 reflections with *I* > 2σ(*I*)
graphite	*R*_int_ = 0.065
ω scans	θ_max_ = 27.7°, θ_min_ = 2.3°
Absorption correction: multi-scan (*SADABS*; Bruker, 2008)	*h* = −7→11
*T*_min_ = 0.976, *T*_max_ = 0.980	*k* = −9→12
10510 measured reflections	*l* = −28→28

### Refinement

**Table:**

Refinement on *F*^2^	Primary atom site location: structure-invariant direct methods
Least-squares matrix: full	Secondary atom site location: difference Fourier map
*R*[*F*^2^ > 2σ(*F*^2^)] = 0.081	Hydrogen site location: inferred from neighbouring sites
*wR*(*F*^2^) = 0.252	H-atom parameters constrained
*S* = 1.00	*w* = 1/[σ^2^(*F*_o_^2^) + (0.1392*P*)^2^ + 0.1028*P*] where *P* = (*F*_o_^2^ + 2*F*_c_^2^)/3
4476 reflections	(Δ/σ)_max_ < 0.001
254 parameters	Δρ_max_ = 0.29 e Å^−^^3^
0 restraints	Δρ_min_ = −0.33 e Å^−^^3^

### Special details

**Table:**

Geometry. All e.s.d.'s (except the e.s.d. in the dihedral angle between two l.s. planes) are estimated using the full covariance matrix. The cell e.s.d.'s are taken into account individually in the estimation of e.s.d.'s in distances, angles and torsion angles; correlations between e.s.d.'s in cell parameters are only used when they are defined by crystal symmetry. An approximate (isotropic) treatment of cell e.s.d.'s is used for estimating e.s.d.'s involving l.s. planes.
Refinement. Refinement of *F*^2^ against ALL reflections. The weighted *R*-factor *wR* and goodness of fit *S* are based on *F*^2^, conventional *R*-factors *R* are based on *F*, with *F* set to zero for negative *F*^2^. The threshold expression of *F*^2^ > σ(*F*^2^) is used only for calculating *R*-factors(gt) *etc*. and is not relevant to the choice of reflections for refinement. *R*-factors based on *F*^2^ are statistically about twice as large as those based on *F*, and *R*- factors based on ALL data will be even larger.

### Fractional atomic coordinates and isotropic or equivalent isotropic displacement parameters (Å^2^)

**Table:**

	*x*	*y*	*z*	*U*_iso_*/*U*_eq_	
C1	0.7119 (5)	0.2084 (4)	0.21685 (18)	0.0428 (9)	
C2	0.7325 (5)	0.0754 (4)	0.2349 (2)	0.0576 (12)	
H2	0.6914	0.0050	0.2123	0.069*	
C3	0.8128 (6)	0.0469 (5)	0.2855 (2)	0.0655 (14)	
H3	0.8273	−0.0425	0.2977	0.079*	
C4	0.8728 (6)	0.1533 (5)	0.3188 (2)	0.0605 (12)	
H4	0.9273	0.1352	0.3537	0.073*	
C5	0.8522 (5)	0.2841 (5)	0.30056 (17)	0.0522 (11)	
H5	0.8937	0.3541	0.3232	0.063*	
C6	0.7705 (4)	0.3161 (4)	0.24889 (16)	0.0414 (9)	
C7	0.7539 (4)	0.4578 (4)	0.22771 (15)	0.0383 (9)	
H7	0.8516	0.5017	0.2259	0.046*	
C8	0.6754 (4)	0.4707 (4)	0.16629 (15)	0.0402 (9)	
C9	0.5624 (5)	0.3556 (4)	0.1614 (2)	0.0533 (11)	
H9A	0.4902	0.3641	0.1938	0.064*	
H9B	0.5097	0.3632	0.1232	0.064*	
C10	0.7823 (6)	0.4613 (5)	0.11672 (17)	0.0520 (11)	
C11	0.5993 (5)	0.6120 (4)	0.17064 (17)	0.0459 (10)	
H11	0.4913	0.5988	0.1703	0.055*	
C12	0.6383 (5)	0.7144 (4)	0.12342 (16)	0.0448 (9)	
C13	0.7633 (6)	0.7942 (5)	0.12838 (19)	0.0609 (13)	
H13	0.8258	0.7839	0.1616	0.073*	
C14	0.7971 (7)	0.8890 (5)	0.0848 (2)	0.0728 (15)	
H14	0.8806	0.9436	0.0897	0.087*	
C15	0.7101 (7)	0.9044 (5)	0.0344 (2)	0.0684 (15)	
C16	0.5876 (7)	0.8215 (7)	0.0288 (2)	0.0774 (17)	
H16	0.5266	0.8291	−0.0051	0.093*	
C17	0.5550 (6)	0.7284 (6)	0.0726 (2)	0.0710 (15)	
H17	0.4727	0.6724	0.0674	0.085*	
C18	0.7427 (10)	1.0141 (8)	−0.0113 (3)	0.112 (3)	
H18A	0.7231	0.9804	−0.0513	0.168*	
H18B	0.8453	1.0404	−0.0083	0.168*	
H18C	0.6805	1.0914	−0.0035	0.168*	
C19	0.7143 (5)	0.5804 (4)	0.32222 (16)	0.0457 (10)	
C20	0.6470 (6)	0.5344 (6)	0.37311 (17)	0.0678 (14)	
H20	0.5608	0.4825	0.3703	0.081*	
C21	0.7058 (8)	0.5643 (8)	0.4288 (2)	0.088 (2)	
H21	0.6590	0.5329	0.4637	0.105*	
C22	0.8317 (9)	0.6392 (7)	0.4334 (2)	0.091 (2)	
H22	0.8712	0.6592	0.4713	0.110*	
C23	0.9005 (8)	0.6853 (6)	0.3823 (2)	0.0874 (19)	
H23	0.9874	0.7361	0.3853	0.105*	
C24	0.8416 (6)	0.6567 (5)	0.3261 (2)	0.0658 (14)	
H24	0.8877	0.6888	0.2912	0.079*	
N1	0.6521 (4)	0.5396 (3)	0.26516 (13)	0.0454 (8)	
N2	0.8669 (6)	0.4523 (5)	0.07901 (16)	0.0717 (13)	
O1	0.6300 (4)	0.2284 (3)	0.16486 (13)	0.0585 (8)	
O2	0.6411 (4)	0.6622 (3)	0.22807 (11)	0.0539 (8)	

### Atomic displacement parameters (Å^2^)

**Table:**

	*U*^11^	*U*^22^	*U*^33^	*U*^12^	*U*^13^	*U*^23^
C1	0.037 (2)	0.033 (2)	0.059 (2)	0.0030 (18)	0.0031 (18)	0.0012 (18)
C2	0.045 (3)	0.030 (2)	0.098 (3)	0.002 (2)	0.007 (3)	0.000 (2)
C3	0.051 (3)	0.041 (3)	0.105 (4)	0.006 (2)	0.014 (3)	0.023 (3)
C4	0.053 (3)	0.054 (3)	0.074 (3)	0.019 (3)	0.000 (2)	0.015 (2)
C5	0.053 (3)	0.046 (2)	0.057 (2)	0.007 (2)	−0.003 (2)	0.003 (2)
C6	0.034 (2)	0.039 (2)	0.0515 (19)	0.0039 (18)	0.0058 (16)	0.0040 (17)
C7	0.036 (2)	0.035 (2)	0.0440 (18)	0.0044 (17)	0.0030 (16)	−0.0010 (17)
C8	0.034 (2)	0.039 (2)	0.0475 (19)	0.0019 (18)	0.0014 (16)	0.0024 (17)
C9	0.048 (3)	0.045 (3)	0.067 (3)	−0.005 (2)	−0.008 (2)	−0.002 (2)
C10	0.066 (3)	0.048 (2)	0.0419 (19)	0.007 (2)	0.002 (2)	0.0000 (19)
C11	0.038 (2)	0.049 (2)	0.051 (2)	0.0084 (19)	−0.0003 (17)	0.0013 (19)
C12	0.041 (3)	0.044 (2)	0.0493 (19)	0.006 (2)	0.0000 (18)	0.0058 (18)
C13	0.060 (3)	0.057 (3)	0.065 (3)	0.002 (3)	−0.011 (2)	0.015 (2)
C14	0.067 (4)	0.057 (3)	0.095 (4)	−0.006 (3)	0.003 (3)	0.019 (3)
C15	0.082 (4)	0.059 (3)	0.064 (3)	0.023 (3)	0.010 (3)	0.020 (3)
C16	0.077 (4)	0.096 (5)	0.059 (3)	0.011 (4)	−0.011 (3)	0.024 (3)
C17	0.066 (4)	0.077 (4)	0.070 (3)	−0.008 (3)	−0.022 (2)	0.012 (3)
C18	0.126 (7)	0.102 (5)	0.108 (4)	0.023 (5)	0.016 (4)	0.055 (4)
C19	0.053 (3)	0.039 (2)	0.046 (2)	0.009 (2)	0.0059 (18)	0.0024 (18)
C20	0.062 (3)	0.091 (4)	0.050 (2)	0.001 (3)	0.009 (2)	0.007 (3)
C21	0.100 (5)	0.116 (6)	0.048 (3)	0.003 (5)	0.009 (3)	0.000 (3)
C22	0.135 (7)	0.083 (4)	0.056 (3)	−0.002 (5)	−0.016 (3)	−0.018 (3)
C23	0.117 (5)	0.068 (4)	0.077 (3)	−0.033 (4)	−0.016 (3)	−0.011 (3)
C24	0.085 (4)	0.052 (3)	0.061 (2)	−0.018 (3)	−0.001 (3)	−0.004 (2)
N1	0.046 (2)	0.0425 (19)	0.0473 (16)	0.0103 (18)	0.0050 (15)	0.0043 (15)
N2	0.076 (3)	0.083 (3)	0.056 (2)	0.017 (3)	0.009 (2)	0.003 (2)
O1	0.065 (2)	0.0405 (17)	0.0696 (18)	−0.0055 (16)	−0.0100 (16)	−0.0047 (14)
O2	0.067 (2)	0.0478 (17)	0.0469 (13)	0.0153 (17)	0.0043 (14)	0.0060 (13)

### Geometric parameters (Å, °)

**Table:**

C1—C6	1.379 (5)	C12—C13	1.376 (6)
C1—O1	1.378 (5)	C13—C14	1.373 (6)
C1—C2	1.381 (5)	C13—H13	0.9300
C2—C3	1.360 (7)	C14—C15	1.369 (7)
C2—H2	0.9300	C14—H14	0.9300
C3—C4	1.388 (7)	C15—C16	1.376 (8)
C3—H3	0.9300	C15—C18	1.507 (7)
C4—C5	1.362 (6)	C16—C17	1.364 (7)
C4—H4	0.9300	C16—H16	0.9300
C5—C6	1.393 (5)	C17—H17	0.9300
C5—H5	0.9300	C18—H18A	0.9600
C6—C7	1.478 (5)	C18—H18B	0.9600
C7—N1	1.473 (5)	C18—H18C	0.9600
C7—C8	1.534 (5)	C19—C20	1.354 (6)
C7—H7	0.9800	C19—C24	1.371 (7)
C8—C10	1.459 (6)	C19—N1	1.436 (5)
C8—C9	1.526 (6)	C20—C21	1.371 (7)
C8—C11	1.553 (6)	C20—H20	0.9300
C9—O1	1.394 (5)	C21—C22	1.355 (10)
C9—H9A	0.9700	C21—H21	0.9300
C9—H9B	0.9700	C22—C23	1.365 (8)
C10—N2	1.131 (6)	C22—H22	0.9300
C11—O2	1.412 (5)	C23—C24	1.377 (7)
C11—C12	1.492 (5)	C23—H23	0.9300
C11—H11	0.9800	C24—H24	0.9300
C12—C17	1.357 (6)	N1—O2	1.461 (4)
			
C6—C1—O1	121.4 (3)	C13—C12—C11	121.5 (4)
C6—C1—C2	122.0 (4)	C12—C13—C14	120.8 (4)
O1—C1—C2	116.6 (4)	C12—C13—H13	119.6
C3—C2—C1	120.3 (4)	C14—C13—H13	119.6
C3—C2—H2	119.9	C15—C14—C13	121.2 (5)
C1—C2—H2	119.9	C15—C14—H14	119.4
C2—C3—C4	119.1 (4)	C13—C14—H14	119.4
C2—C3—H3	120.5	C14—C15—C16	117.7 (5)
C4—C3—H3	120.5	C14—C15—C18	120.9 (6)
C5—C4—C3	120.2 (4)	C16—C15—C18	121.3 (6)
C5—C4—H4	119.9	C17—C16—C15	120.4 (5)
C3—C4—H4	119.9	C17—C16—H16	119.8
C4—C5—C6	121.9 (4)	C15—C16—H16	119.8
C4—C5—H5	119.1	C12—C17—C16	122.5 (5)
C6—C5—H5	119.1	C12—C17—H17	118.8
C1—C6—C5	116.6 (4)	C16—C17—H17	118.8
C1—C6—C7	121.6 (3)	C15—C18—H18A	109.5
C5—C6—C7	121.7 (4)	C15—C18—H18B	109.5
N1—C7—C6	113.6 (3)	H18A—C18—H18B	109.5
N1—C7—C8	99.5 (3)	C15—C18—H18C	109.5
C6—C7—C8	113.8 (3)	H18A—C18—H18C	109.5
N1—C7—H7	109.8	H18B—C18—H18C	109.5
C6—C7—H7	109.8	C20—C19—C24	120.2 (4)
C8—C7—H7	109.8	C20—C19—N1	117.5 (4)
C10—C8—C9	109.8 (4)	C24—C19—N1	122.2 (4)
C10—C8—C7	110.8 (3)	C19—C20—C21	120.1 (6)
C9—C8—C7	107.9 (3)	C19—C20—H20	120.0
C10—C8—C11	113.1 (3)	C21—C20—H20	120.0
C9—C8—C11	112.1 (3)	C22—C21—C20	120.4 (5)
C7—C8—C11	102.8 (3)	C22—C21—H21	119.8
O1—C9—C8	111.9 (4)	C20—C21—H21	119.8
O1—C9—H9A	109.2	C23—C22—C21	119.8 (5)
C8—C9—H9A	109.2	C23—C22—H22	120.1
O1—C9—H9B	109.2	C21—C22—H22	120.1
C8—C9—H9B	109.2	C22—C23—C24	120.2 (6)
H9A—C9—H9B	107.9	C22—C23—H23	119.9
N2—C10—C8	178.6 (5)	C24—C23—H23	119.9
O2—C11—C12	109.2 (3)	C19—C24—C23	119.3 (5)
O2—C11—C8	104.6 (3)	C19—C24—H24	120.3
C12—C11—C8	117.3 (3)	C23—C24—H24	120.3
O2—C11—H11	108.5	C19—N1—O2	106.7 (3)
C12—C11—H11	108.5	C19—N1—C7	113.8 (3)
C8—C11—H11	108.5	O2—N1—C7	100.3 (3)
C17—C12—C13	117.3 (4)	C1—O1—C9	114.0 (3)
C17—C12—C11	121.2 (4)	C11—O2—N1	103.5 (3)
			
C6—C1—C2—C3	−0.1 (7)	C17—C12—C13—C14	3.3 (7)
O1—C1—C2—C3	179.6 (4)	C11—C12—C13—C14	−179.4 (5)
C1—C2—C3—C4	0.3 (7)	C12—C13—C14—C15	−1.8 (8)
C2—C3—C4—C5	−0.5 (8)	C13—C14—C15—C16	−0.1 (8)
C3—C4—C5—C6	0.4 (8)	C13—C14—C15—C18	176.3 (5)
O1—C1—C6—C5	−179.6 (3)	C14—C15—C16—C17	0.5 (9)
C2—C1—C6—C5	0.1 (6)	C18—C15—C16—C17	−176.0 (6)
O1—C1—C6—C7	−2.6 (6)	C13—C12—C17—C16	−3.0 (8)
C2—C1—C6—C7	177.1 (4)	C11—C12—C17—C16	179.7 (5)
C4—C5—C6—C1	−0.2 (6)	C15—C16—C17—C12	1.2 (9)
C4—C5—C6—C7	−177.2 (4)	C24—C19—C20—C21	0.2 (8)
C1—C6—C7—N1	109.5 (4)	N1—C19—C20—C21	176.7 (5)
C5—C6—C7—N1	−73.6 (5)	C19—C20—C21—C22	−0.3 (10)
C1—C6—C7—C8	−3.5 (5)	C20—C21—C22—C23	0.0 (11)
C5—C6—C7—C8	173.4 (3)	C21—C22—C23—C24	0.5 (10)
N1—C7—C8—C10	150.9 (3)	C20—C19—C24—C23	0.3 (8)
C6—C7—C8—C10	−87.8 (4)	N1—C19—C24—C23	−176.0 (5)
N1—C7—C8—C9	−88.8 (4)	C22—C23—C24—C19	−0.7 (9)
C6—C7—C8—C9	32.4 (4)	C20—C19—N1—O2	132.9 (4)
N1—C7—C8—C11	29.8 (4)	C24—C19—N1—O2	−50.7 (5)
C6—C7—C8—C11	151.0 (3)	C20—C19—N1—C7	−117.4 (4)
C10—C8—C9—O1	60.6 (4)	C24—C19—N1—C7	59.0 (5)
C7—C8—C9—O1	−60.3 (4)	C6—C7—N1—C19	75.1 (4)
C11—C8—C9—O1	−172.8 (3)	C8—C7—N1—C19	−163.5 (3)
C10—C8—C11—O2	−118.3 (4)	C6—C7—N1—O2	−171.3 (3)
C9—C8—C11—O2	116.9 (4)	C8—C7—N1—O2	−49.9 (3)
C7—C8—C11—O2	1.3 (4)	C6—C1—O1—C9	−24.7 (5)
C10—C8—C11—C12	2.8 (5)	C2—C1—O1—C9	155.6 (4)
C9—C8—C11—C12	−122.0 (4)	C8—C9—O1—C1	57.2 (5)
C7—C8—C11—C12	122.4 (4)	C12—C11—O2—N1	−158.9 (3)
O2—C11—C12—C17	−147.9 (4)	C8—C11—O2—N1	−32.6 (4)
C8—C11—C12—C17	93.4 (5)	C19—N1—O2—C11	172.2 (3)
O2—C11—C12—C13	34.9 (5)	C7—N1—O2—C11	53.3 (4)
C8—C11—C12—C13	−83.8 (5)		

### Hydrogen-bond geometry (Å, °)

**Table:**

Cg1 and Cg2 are the centroids of the C19–C24 and C12–C17 rings, respectively.

**Table:**

*D*—H···*A*	*D*—H	H···*A*	*D*···*A*	*D*—H···*A*
C5—H5···Cg1	0.93	2.99	3.701 (5)	134
C20—H20···Cg2^i^	0.93	2.95	3.803 (6)	153
C21—H21···N2^ii^	0.93	2.56	3.385 (6)	148
C23—H23···N2^iii^	0.93	2.62	3.466 (7)	151

Symmetry codes: (i) −*x*+1, *y*−1/2, −*z*+1/2; (ii) −*x*+3/2, −*y*+1, *z*+1/2; (iii) −*x*+2, *y*+1/2, −*z*+1/2.
